# Deflection test evaluation of different lots of the same nickel-titanium wire commercial brand

**DOI:** 10.1590/2177-6709.21.1.042-046.oar

**Published:** 2016

**Authors:** Murilo Gaby Neves, Fabrício Viana Pereira Lima, Júlio de Araújo Gurgel, Célia Regina Maio Pinzan-Vercelino, Fernanda Soares Rezende, Gustavo Antônio Martins Brandão

**Affiliations:** 1 MSc in Orthodontics, Universidade Ceuma, São Luís, Maranhão, Brazil; 2 Professor, Universidade Ceuma, Masters Program in Dentistry, São Luís, Maranhão, Brazil; 3 Undergraduate student in Dentistry, Universidade Ceuma, São Luís, Maranhão, Brazil; 4 Adjunct professor III of Dentistry, Universidade Federal do Pará (UFPA), School of Dentistry, Belém, Pará, Brazil

**Keywords:** Elasticity, Physical properties, Orthodontic wires

## Abstract

**Introduction:**

The aim of this *in vitro* study was to compare the elastic properties of the load-deflection ratio of orthodontic wires of different lot numbers and the same commercial brand.

**Methods:**

A total of 40 nickel-titanium (NiTi) wire segments (Morelli OrtodontiaTM - Sorocaba, SP, Brazil), 0.016-in in diameter were used. Groups were sorted according to lot numbers (lots 1, 2, 3 and 4). 28-mm length segments from the straight portion (ends) of archwires were used. Deflection tests were performed in an EMIC universal testing machine with 5-N load cell at 1 mm/minute speed. Force at deactivation was recorded at 0.5, 1, 2 and 3 mm deflection. Analysis of variance (ANOVA) was used to compare differences between group means.

**Results:**

When comparing the force of groups at the same deflection (3, 2 and 1 mm), during deactivation, no statistical differences were found.

**Conclusion:**

There are no changes in the elastic properties of different lots of the same commercial brand; thus, the use of different lots of the orthodontic wires used in this research does not compromise the final outcomes of the load-deflection ratio.

## Abstract (pt)

**Introdução:**

o objetivo deste estudo experimental *in vitro* foi comparar a as propriedades elásticas da relação carga/deflexão para fios de diferentes lotes de uma mesma marca comercial.

**Métodos:**

foram selecionados quarenta segmentos de arcos de níquel-titânio superelástico (NiTi) (Morelli Ortodontia, Sorocaba/SP, Brasil), com diâmetro de 0,016". Os grupos foram ordenados de acordo com os lotes (lotes 1, 2, 3 e 4). Foram utilizados segmentos com 28mm de comprimento da parte reta (extremidades) dos arcos pré-contornados. Os testes de deflexão foram realizados em uma máquina universal de ensaios EMIC, com célula de carga de 5N e velocidade de 1mm/minuto. A força durante a desativação foi registrada nas deflexões de 0,5; 1; 2 e 3mm. O teste de Análise da Variância (ANOVA) foi utilizado para comparar diferenças entre as médias dos grupos.

**Resultados:**

na comparação de força dos lotes em um mesmo ponto de deflexão (3, 2 e 1mm), durante a desativação, não houve diferenças estatísticas.

**Conclusões:**

não ocorre alteração nas propriedades elásticas em diferentes lotes da mesma marca. Assim, o uso de diferentes lotes dos fios ortodônticos utilizados nesta pesquisa não comprometeu o resultado final da relação carga/deflexão.

## INTRODUCTION

The alignment-leveling phase is characterized by progressive increase in wire diameter. Initially, nickel-titanium (NiTi) wires are used, followed by stainless steel wires used at the final stages of treatment, always respecting the periodontal physiological limits and prediction of expected results.^1^ NiTi 0.016-in round wires promote partial alignment and leveling of teeth at the early stages of treatment.^2^^,^ 3 ^,^ 4

Extensive knowledge about the mechanical properties of orthodontic wires is crucial, and wires must be selected according to their behavior.^5^^,^ 6 ^,^ 7 The advent of ISO for orthodontic wires enhanced our understanding and comparison between wires manufactured with different metal alloys as well as wires of different commercial brands. Thus, manufacturers are able to better arrange the real characteristics of their products.^8^

Presently, one of the most reliable and clinically applicable means of evaluating orthodontic wires is with the aid of deflection tests. It satisfactorily simulates what really occurs in clinical practice when placing a wire into the bracket slot.^9^

ISO tests^10^ allowed the physical properties of orthodontic wires of different lots produced by the same manufacturer to be standardized. It is expected that the wires present the physical properties described on their labels. Thus, it seems appropriate to submit wires to tests that validate their properties,^11 ^regardless of the lot used.

An experimental *in vitro* study was conducted to investigate potential changes in the physical properties in different lots of the same commercial brand of orthodontic wires, with potential effects on their elastic properties (load-deflection ratio). The null hypothesis tested was that there are no changes in the elastic properties in different lots of orthodontic wires of the same commercial brand.

## MATERIAL AND METHODS

The sample comprised 40 segments of conventional NiTi round wires, 0.016-in in diameter (Morelli Ortodontia^TM^, Sorocaba, São Paulo, Brazil). Each group comprised 10 archwire segments, arranged according to the lots. Group 1 (lot 1) had 55.87% of nickel in its composition; group 2 (lot 2) had 55.95% of nickel; group 3 (lot 3) had 56.00% of nickel; and group 4 (lot 4) had 55.96% of nickel in its composition. All wires were manufactured during the same period and were subject to the same storage conditions: dry environment and at room temperature.

Specimens were obtained by cutting 28-mm segments from the straight portion (ends) of orthodontic precontoured archwires. Each arch provided one specimen. Specimens were segmented by the same individual with the aid of a light wire plier and a digital caliper used to measure the segments.^13^

During trials, specimens were secured by means of a metallic device consisting of a support and two 5-mm diameter pins welded vertically 14 mm apart.^10^ A Morelli Ortodontia^TM^, Edgewise prescription, 0.022-in premolar metal bracket was attached to one of the pins, and a standard molar tube of the same brand was attached to the other pin. Both pieces were fixed with Orthobond resin (Morelli Ortodontia^TM)^. For better alignment during fixing of orthodontic devices, a segment of stainless steel 0.021 x 0.025-in wire and elastic ligature in modules (Morelli Ortodontia^TM)^ were used.

A digital caliper (150-mm Coolant Proof Absolute, Mitutoyo, Aurora, Illinois, USA) was used to aid bonding and measure interbracket distance, previously determined at 14 mm. 

Deflection tests were carried out to evaluate the load-deflection ratio of orthodontic wires. To this end, a universal testing machine (EMIC, DL2000 model), with 5-N load cell and at a speed of 1 mm/minute, was used. The three-point bending test was employed due to best simulating orthodontic clinical practice.^8^^,^ 12 Force applied during deactivation was registered at deflections 0.5, 1, 2 and 3 mm.

Analysis of variance (ANOVA) was employed to compare differences between group means for the deflection test force. This is because data were parametric and presented with equality and normality of variances (heteroscedasticity).

## RESULTS

Table 1 shows descriptive statistics results in relation to deactivation expressed in Newton (N) for the groups tested. According to ANOVA (one-way), there were no statistical differences between forces applied to lots under the same deflection (3, 2 or 1 mm) during deactivation (Table 1). Thus, all lots showed similar load-deflection ratios at given deactivation points. When an inter-relationship between forces is established at deflections 0.5, 1.0, 2.0 and 3.0 mm, during deactivation, statistical differences are found among all deflection measures. Thus, there is no such thing as an ongoing force, which mischaracterizes the formation of an unloading plateau. Table 2 described the comparison between a given deflection and all other deflection values of a given wire (Table 2).

**Table 1:** - Descriptive analysis of variables in relation to deactivation, with mean force values (N) and deflection standard deviation (mm) within a 3.0 to 0.5-mm interval. *P* value for comparison between groups at the same deflection.

**Groups**		**Deactivation values**			
	**Deformation**	**3.0 mm**	**2.0 mm**	**1.0 mm**	**0.5 mm**
Lot 1	Mean (SD)	2.78 (1.05)	1.78 (0.57)	1.24 (0.43)	0.61 (0.25)
Lot 2	Mean (SD)	3.32 (0.65)	1.98 (0.31)	1.30 (0.16)	0.69 (0.26)
Lot 3	Mean (SD)	3.38 (0.26)	2.10 (0.21)	1.47 (0.16)	0.78 (0.31)
Lot 4	Mean (SD)	3.57 (0.31)	1.96 (0.19)	1.28 (0.16)	0.69 (0.34)
p value	0.0603	0.2663	0.2094		

**Table 2:** - Inter-relationship between deflection values at deactivation.

**(I) Deflection**	**(J) Deflection**	**Mean difference (I-J)**
0.5 mm	1 mm	-0.6300
	2 mm	-1.2625
	3 mm	-2.5700
1 mm	0.5 mm	0.6300
	2 mm	-0.6325
	3 mm	-1.9400
2 mm	0.5 mm	1.2625
	1 mm	0.6325
	3 mm	-1.3075
3 mm	0.5 mm	2.5700
	1 mm	1.9400
	2 mm	1.3075

## DISCUSSION

Knowing the load-deflection ratio of a given orthodontic wire allows wires to be better selected for treatment - particularly at the alignment-leveling phase, during which wire deactivation and tooth movement occur. Understanding the force applied enhances orthodontic movement, thereby preventing side effects.

NiTi archwires are popular because of their properties and potential for movement under light and continuous forces, which allows tooth movement by means of more adequate forces.^14^ In order to achieve greater predictability of forces released by orthodontic wires, laboratory tests are recommended to assess elastic properties.^14^^,^ 15

Although orthodontic wires of the same model, diameter and features undergo the same manufacturing process, they present with changes in their composition that vary according to the lot, as observed in the samples of the present study. Therefore, for some authors,^16^ using a set of wires of the same lot eliminates selection bias in studies; in other words, the influence exerted by the lot over research outcomes is eliminated. That is the reason why researches are concerned about this type of standardization. Nevertheless, such scientific consensus has not been proved and is used arbitrarily to pass on the idea of a reliable study.

In order to achieve more reliable outcomes in researches using material, whether as the major element of investigation or as part of a given study, the ideal is to use material of the same commercial brand and lot. The results yielded by a study conducted by Sakima et al^16^ highlight the importance of laboratory trials to assess the elastic properties of wires, revealing that the loading and unloading curve in the load-deflection ratio chart might vary between wires, even though those wires are of the same type and manufacturer.^17^^,^ 18 Of the factors affecting such variation, the authors point out that the lot might influence the outcomes.^19^

Nevertheless, the major finding of the present investigation proved to be of considerable laboratory relevance. This is because when the forces of different lots were compared for the same deflection point at deactivation, no statistical differences were found: 1 mm with *p* = 0.2094; 2 mm with*p* = 0.2663; 3 mm with *p* = 0.0603. These outcomes justify the use of orthodontic wires of the same commercial brand but of different lots, without hindering the final results of the study (Table 1).

Even though the manufacturer of the wires assessed herein describe them as "NiTi superelastic", which characterizes a plateau region in the load-deflection ratio chart, when the forces between deflection values of a given plateau were compared, no statistically steady forces were found during deactivation. Thus, a greater slope, or a parameter of force loss, was found at deactivation. This represents a significant difference between the initial force at the plateau region and the final force at this region of the chart curve.^4^^,^ 14

Orthodontic wire superelasticity produces a relatively steady force within a given deactivation interval, the phase during which the wire attached to an orthodontic device induces tooth movement.^20 ^Unlike the present research, lack of steady force at deactivation mischaracterizes the superelasticity proposed by the wire manufacturer, which could produce a less predictable or less biological movement during clinical practice. 

Thus, it is suggested that researches using wires of the same commercial brand used in the present study do not require lots to be standardized, so as to yield more reliable results, even though other authors have determined that wires of the same lot be used.^21^^,^ 22 Nevertheless, further studies are warranted to assess wires of different models, diameters and manufacturers.

## CONCLUSIONS

Based on the results of the present study, it is reasonable to conclude that there were no changes in the elastic properties in different lots of wires of the same commercial brand. Therefore, using different lots of the orthodontic wires used in the present research does not hinder the final outcomes of the load-deflection ratio.
